# A refractory heterogeneous Cryopyrin-Associated Periodic Syndrome (CAPS) phenotype related to *V198M* mutation responds to canakinumab - a case report

**DOI:** 10.1186/1546-0096-9-S1-P9

**Published:** 2011-09-14

**Authors:** Sandra Hansmann, Christiane Zimmer, Monika Moll, Nikolaus Rieber, Jasmin B Kuemmerle-Deschner

**Affiliations:** 1 Division of Pediatric Rheumatology, Department of Pediatrics, University Hospital Tübingen, Germany

## Background

*V198M* mutation has been identified in more than one CAPS phenotype and is difficult to treat.

## Aim

To report effects of prolonged selective IL-1β inhibition with canakinumab in a CAPS phenotype related to *V198M* mutation.

## Methods

A 9-year old boy, diagnosed with symptomatic MWS and evidence of a *V198M* mutation (father also *V198M*), only partially responsive to anakinra, participated in a phase III study of canakinumab in CAPS patients (N=166). Study treatment was 8-weekly administration of canakinumab by s.c. injection, 150 mg to adults and 2 mg/kg to patients ≤40 kg for up to 2 years. Administration frequency and dose (maximum 600 mg or 8 mg/kg [≤40 kg]) were increased in case of residual symptoms.

## Results

After insufficient response to per-protocol canakinumab dose, the patient received high dose canakinumab (10 mg/kg, i.v.) every 4 weeks from Day 20 onwards to >440 days. Disease activity improved from severe to moderate at Day 48 and remained stable including normal CRP and SAA values (<10 mg/L). Temporary mild adverse events (AE) were not suspected to be treatment-related (upper respiratory viral infection, gastroenteritis, and rhinitis). Night sweats, dry lips and skin persisted until last assessments. No serious AEs /infections were reported in this patient.

## Conclusion

Based on our experience in this patient and a review of literature on efficacy of IL-1 inhibition in CAPS patients with *V198M* mutation, high dose canakinumab i.v* with increased dosing frequency yields symptomatic relief without evidence of increased AE. Confirmation of our observation in more patients with similar genetic and clinical presentation is needed.

*The posology used for this case is not in line with the approved posology of canakinumab

